# Comparison of educational performance between the only children and children in two-child families

**DOI:** 10.1038/s41598-022-19730-3

**Published:** 2022-09-12

**Authors:** Yehui Lao, Suxu Lin

**Affiliations:** 1 College of Economy and Trade, Zhongkai University of Agriculture and Engineering, Guangzhou, Guangdong Province China; 2 Guangdong Polytechnic Normal University, Guangzhou, Guangdong Province China

**Keywords:** Psychology, Human behaviour

## Abstract

This paper estimates the causal effect of only-child status on educational performance among junior high school students from only-child and two-child families in China. It uses the dataset of the China Education Panel Survey 2013–14. The results show that the only children's educational outcomes are significantly low than students from two-child families. Only children's willpower and extraversion are weaker than children's from two-child families. The scale economies effect is strong and the resource dilution effect is weak when sibling size is small.

## Introduction

Following the lead of Becker and Lewis^1^, economists have studied the effect of family size on educational attainment, including years engaged in schooling and level if education completed. Based on this research, one well-established opinion is that, because they receive fewer educational investments, children in large families may not attain high level of education^2–5^. Becker and Lewis's^1^ theory predicts a negative family size effect on educational attainment. Many studies follow and extend their theory. Social scientists^6,7^ have revealed that family size has a negative effect on the educational performance of adults, primarily caused by the dilution of resources that accompanies family growth^6^. Additionally, the marginal cost of each additional sibling can amount to a loss of one fifth of a year of schooling^8^. Increasing family size also lowers the share of financial resources for each child, as well as benefits that are transferred across generations, such as their inheritance. Some evidence^9,10^ supports that this effect on children can be evidenced both immediately and over time. Using a provincial sample, Chen et al.^11^ indicated that sibship size has significant effects on decreasing high school and college enrollment. However, Åslund and Grönqvist’s^12^ study showed that sibship size was a minor but significant negative impact on grades in compulsory and secondary school among children who might be vulnerable to further restriction of parental investment. They did not find any evidence of a causal effect on long-term outcomes. Furthermore, family size also has a negative effect on parents’ labor market outcomes. Angrist and Evans^13^ used twins as an instrumental variable for identifying the causality of family size on parents’ income and labor market participation. They found that the incomes of both parents and their labor market participation decreased when the family size increased, which might have a negative impact on their children’s educational performance. Parents invest resources, including financial resources, time, and effort, in their children. Parents' resources are relevant to children's general skill development. Increasing the number of children in a family may dilute the parents' ability to invest meaningfully in each child's human capital. This may lead to a child's low educational attainment.

While a number of studies find that sibship size may have a negative effect on children’s performance, there are still differing opinions regarding this. Steelman et al.^14^ suggested that not all parents' resources were associated with improved children's educational outcomes. Frenette^15^ mentioned that the current literature had not yet reached a consensus on the association between parental investment in child quality and a child's cognitive ability. Furthermore, the family size effect on educational attainment is uncertain^16^. In fact, an additional child may not cause resource dilution. Diaz and Fiel^17^ suggested that a sibling had little effect on the educational attainment of older siblings in families having fewer than five children. The extent of the sibship effect seems to weaken over time in Asian countries^18,19^. Thus, there may be economies of scale relative to raising more children. Theoretically, the scale economy effect should be stronger at smaller quantities^20^. Effective sibling interactions may help overcome the shortage of resource dilution, and parents may gain experience from raising older children, which can in turn help them rear their younger children more effectively. Parents may also learn to prevent the dilution of resources that are the keys to children's educational success^15,21^, Lafortune and Lee^22^.

This study focuses on the only-child effect on student educational attainment. Specifically, we examine the difference in test scores in each subject, such as Chinese, mathematics, and English, between children in one-child families and two-child families. Most previous literature obtained causal estimates by looking at multiple births. They usually estimated the effects of family size from 2 to 3 (such as^3,23^ or 3 to 4^11^. However, it is less clear what happens when the family size goes from 1 to 2. In other labor market contexts, Vladasel^24^ found that negative marginal family size effects for incorporated entrepreneurship on all offspring in families with five or more children. His finding implies that the negative marginal effects do not work until the family size is larger than five children. In education contexts, our results may be similar to Vladsel’s^24^ finding. The potential contribution of this paper is that it may provide a precise estimation of the marginal effect of family size on educational attainment in families of one child or more than one child.

To obtain the causal effect of only children on educational performance, we apply the random gender of the firstborn as an exogenous instrument for whether a student is an only child. Lee^25^ first introduced the "first girl" as an instrument for family size in South Korea. Since both China and South Korea belong to the Confucian cultural circle, this instrument is also applicable to China. The results show that children in one-child families have significantly lower test scores than children in two-child families, even after controlling for potential confounders reflecting students' characteristics and family background. An inverted U-shaped relationship can be expected, as the scale economies effect dominates when there are a small number of children in a family.

### Data

We used a dataset from the China Education Panel Survey (CEPS) for our empirical analysis. The CEPS contains information on students, parents, classrooms, and schools. It is a school-based and nationally representative survey, including 438 classrooms in 112 schools in 28 county-level units in mainland China. We examined the dataset for the 2013–2014 academic year, which includes information for over 20,000 students in seventh and ninth grades.

Since this paper aims to compare the difference in educational performance for children from one-child and two-child families, we used samples from one-child and two-child families in the baseline regression. We also used children from one-child and three-child families as a sample in the heterogeneity analysis, whereas we used compliers as a sample in causality.

**Figure 1:**
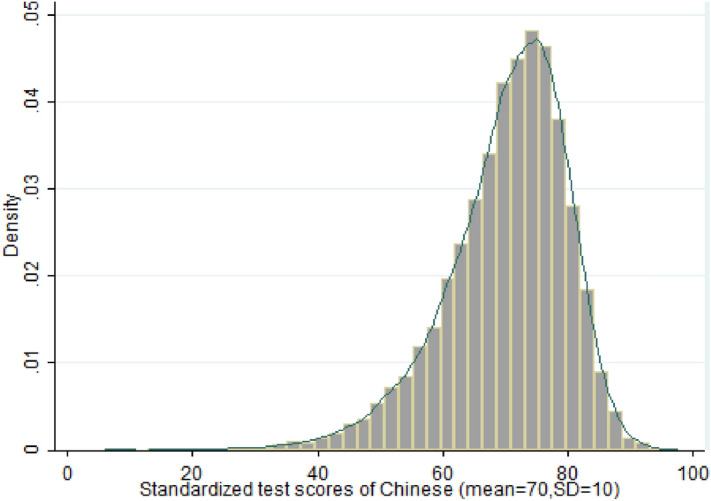
Distribution of Chinese test score.

**Figure 2:**
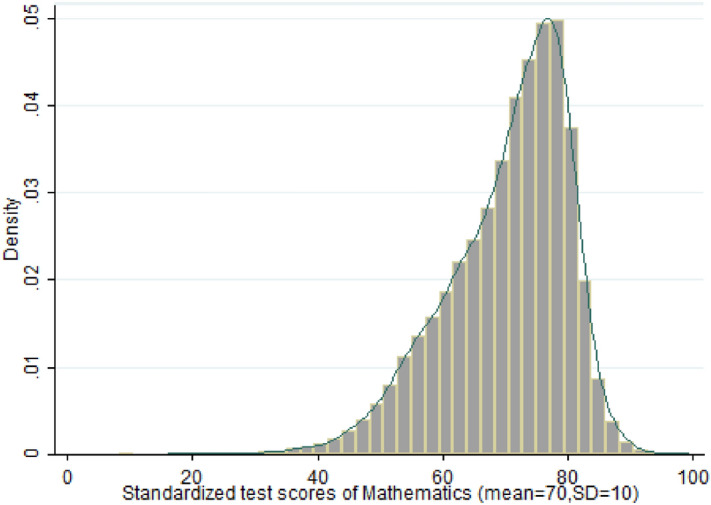
Distribution of mathematics test score.

**Figure 3:**
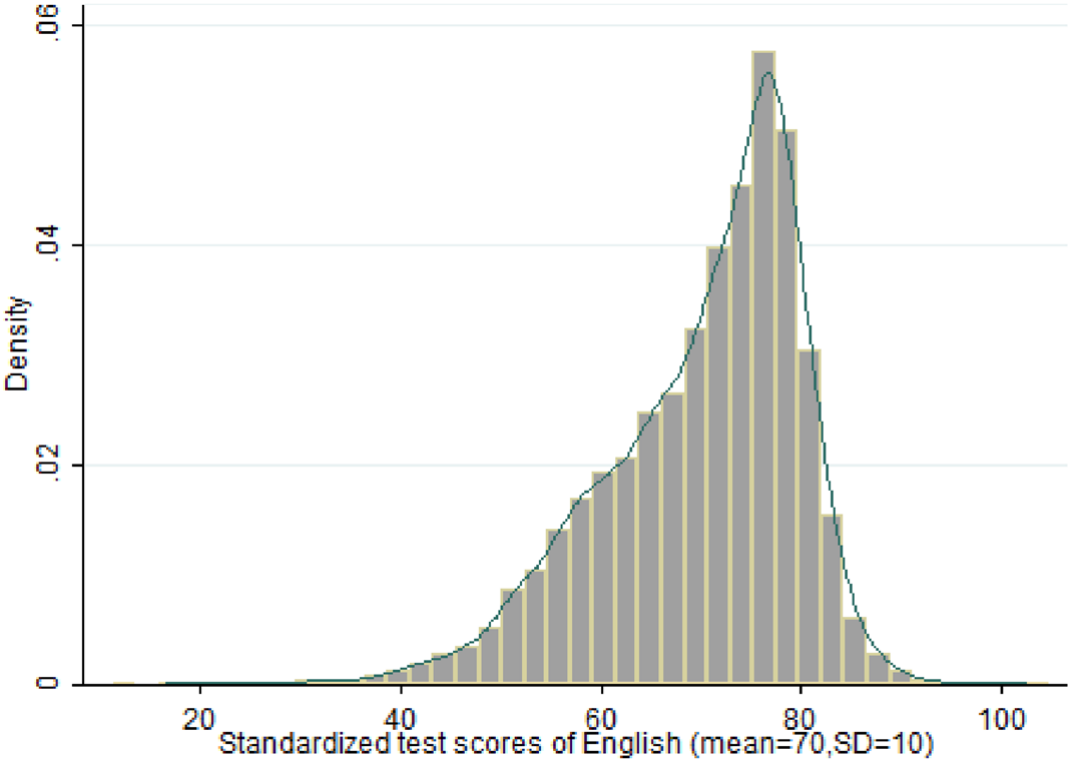
Distribution of English test score.

In addition to a baseline survey on students' personal, family, classroom, and school characteristics, the dataset also contains their standardized test scores in mathematics, Chinese, and English from the fall term. The mean value of the standardized scores is 70, while the standard deviation is 10. The distributions of variables are summarized in Column (1) of Table 1. Figures 1, 2, 3 show the distributions in the Chinese, mathematics and English scores.

**Table 1:** Summary statistics.

	Obs	Mean	Std. Dev
**Outcome variables:**			
Chinese	14,261	70.105	9.841
Math	14,248	70.331	9.733
English	14,252	70.158	9.814
Regressor of interest:			
only_child	14,576	0.580	0.494
**Mediator variables:**			
"I still go to school even I feel a little uncomfortable or have any absent excuses"	14,129	3.301	0.865
"I will try my best to finish my homework even if I do not like it"	14,089	3.288	0.824
"I will try my best to finish my homework even if I have to spend a great deal of time on it"	14,078	3.356	0.813
"How strict your parents are with your homework and exams"	14,534	2.495	0.548
"I consider myself easy to get along with"	14,453	3.194	0.833
"I often take part in school/class activities"	14,434	2.811	1.000
"I often take part in school/class activities"	14,334	2.979	0.916
"How strict are your parents with your school performance"	14,520	2.340	0.599
"How strict are your parents with your school activities"	14,511	2.411	0.659
"How strict are your parents with regarding curfew"	14,491	2.361	0.635
"How strict are your parents regarding your time on the internet"	14,435	2.589	0.599
"How strict are your parents regarding your time watching television"	14,524	2.359	0.658
"How frequently do your parents check your homework and assignment"	14,453	2.482	1.183
"How frequently do your parents tutor you while working on homework and assignment"	14,336	2.094	1.128
**Characteristics:**			
Grade 9	14,576	0.459	0.498
ethnicity (Han)	14,549	0.938	0.241
Gender (male)	14,576	0.539	0.498
Age	14,297	13.414	1.201
**Economics status (ref. group:1)**			
2	13,946	0.186	0.389
3	13,946	0.718	0.450
4	13,946	0.049	0.216
5	13,946	0.002	0.046
**Hukou_at_birth (ref. group:rural)**			
Non-rural	13,826	0.300	0.458
Urban	13,826	0.157	0.364
Other	13,826	0.002	0.048
**Father education**			
Primary school	14,543	0.121	0.326
Middle School	14,543	0.414	0.493
Secondary Vocational school	14,543	0.068	0.251
Vocational high school	14,543	0.024	0.153
High school	14,543	0.179	0.383
College	14,543	0.073	0.260
University	14,543	0.100	0.300
Master above	0.017878	0.133	0.000
**Mother education**			
Primary school	14,543	0.170	0.376
Middle School	14,543	0.408	0.491
Secondary Vocational school	14,543	0.071	0.258
Vocational high school	14,543	0.022	0.145
High school	14,543	0.147	0.354
College	14,543	0.068	0.252
University	14,543	0.082	0.274
Master above	14,543	0.011	0.106

The regressor of interest (only_child) is a dummy variable. Respondents from one-child families have a value of 1, whereas respondents from two-child families (or three-child families, in heterogeneity analysis) have a value of 0. Table 1 shows that children in one-child families accounted for more than half of the sample (58%).

We use willpower, parental control, and educational expectations from parents as mediators in the mechanism check. Table 1 also shows the description of mediator variables.

First, we include three items about self-control to measure willpower, "I still go to school even if I feel a little uncomfortable, and I have not had any absent excuses", "I will try my best to finish my homework even if I do not like it", and "I will try my best to finish my homework even if I have to spend a great deal of time on it". These statements are designed to gauge the willpower of an individual resisting temptation, demonstrating impulse control and overcoming procrastination. This represents the three dimensions of willpower "I Will", "I Won't" and "I Want". To specify, the numerical value 1–4 represents the lowest willpower to the highest ("strongly disagree", "disagree", "agree", and "strongly agree").

Second, we included three items to measure the extraversion of a student, "I consider myself easy to get along with", "I feel close to people in this school", and "I often take part in school/class activities" The answer choice were "strongly disagree", "disagree", "agree" and "strongly agree". All variables are intended to measure students’ extraversion.

Third, we include three items regarding the strictness if parents to measure parental control. They are "how strict are your parents concerning your homework and exams", "how strict your parents are with your school performance", "how strict are your parents concerning your school activities", "how strict are your parents regarding curfew", "how strict are your parents regarding your time using the Internet", and "how strict are your parents regarding your time spent watching television". The numerical value 1–3 represents the parents' strictness from lowest to highest ("1-not strict", "2-normal", and "3-strict").

Finally, we include two items related to parental support, "How frequently do your parents check your homework and assignments?" (check) and "How frequently do your parents tutor you while working on homework assignments?" (tutor). The numerical value 1–4 represents the lowest parental support to the highest ("never", "1–2 days per week", "3–4 days per week ", and "everyday").

### Empirical strategy and results

#### Baseline regression

Our interest is in determining whether there is an association between a student’s standardized test scores (Score) and being from a one-child family exploring the influence of personal characteristics and family characteristics. We estimate the following model using the ordinary linear squared method (OLS):

1 $$Score_{igc} = \beta_{0} + \beta_{1} \times Only\_Child_{igc} + X\beta + Grade + Classroom + \varepsilon_{igc}$$

where ${Score}_{igc}$ represents scores of a grade g student i from classroom c. We compare the scores of children in one-child families to children in two-child families. If the value of ${Only\_Child}_{igc}$ was 1, student i was from a one-child family; otherwise, the student i was from a two-child family. The set of control variables X includes student characteristics (i.e., ethnicity, gender, age, and age squared) and family characteristics (i.e., economic status, hukou type at birth, parental education). The Hukou is Chinese household registration system. The system descends in part from ancient Chinese household registration systems. The hukou system also influenced similar systems in neighboring East Asian countries—such as one within the public administration structures of Japan (koseki) and Korea (hoju), as well as Vietnam (hộ khẩu). The functions of the system regulate population distribution. In mainland China, all PRC nationals’ personal hukou status is classified by residential location (rural/non-rural/urban). The hukou status always connects to individual’s benefits. For example, rural residents have right to receive benefits from Village-owned Enterprise and land rent of their village. $Grade$ is the grade fixed effect, and $Classroom$ is the classroom fixed effect. For ease of interpretation, all scores are standardized to have a mean of 70 and a standard deviation of 10.

Table 2 reports the results of estimating Eq. (1) for the standardized scores of Chinese, mathematics, and English. Columns (1), (3), and (5) report results without any controls, whereas Columns (2), (4), and (6) include the full set of control variables. For Chinese students, the predictions show that being an only child has a statistically significant negative effect on exam performance. Specifically, only child Chinese exam scores are 0.54 lower than children from two-child families. For mathematics, only child exam scores are 0.633 lower than the children from two-child families. However, the result of Column (3) is insignificant. The negative effect may be hidden by other confounding factors. For English, the only child effects are insignificant. We also show results of correlation matrix in Tables A1, A2, A3 in Appendix.

**Table 2:** Associations between only-child status and standardized test scores.

	Chinese	Chinese	Math	Math	English	English
	(1)	(2)	(3)	(4)	(5)	(6)
Only_child	− 0.446**	− 0.540**	− 0.131	− 0.633***	− 0.032	− 0.305
	(0.204)	(0.221)	(0.202)	(0.222)	(0.211)	(0.210)
Ethnicity (Han)		− 0.490		0.020		− 0.496
		(0.451)		(0.471)		(0.410)
Gender (male)		− 5.418***		− 0.717***		− 5.102***
		(0.182)		(0.187)		(0.173)
Age		− 2.504		− 4.808***		− 4.453***
		(1.745)		(1.720)		(1.664)
Age_squared		0.066		0.126**		0.118*
		(0.065)		(0.063)		(0.062)
Economics status	Yes	Yes	Yes	Yes	Yes	Yes
Hukou_at_birth	Yes	Yes	Yes	Yes	Yes	Yes
Father education	Yes	Yes	Yes	Yes	Yes	Yes
Mother education	Yes	Yes	Yes	Yes	Yes	Yes
Grade fixed effect	Yes	Yes	Yes	Yes	Yes	Yes
Classroom fixed effect	Yes	Yes	Yes	Yes	Yes	Yes
Observations	14,261	13,179	14,248	13,171	14,252	13,170
R^2^	0.001	0.182	0.000	0.108	0.000	0.192

Notes: Standard errors in parentheses, adjusted for within-classroom clustering. *p < 0.10. **p < 0.05. ***p < 0.01.

Let us now turn to the heterogeneity analysis. Table 3 shows heterogeneous effects by gender. We consider the interaction terms of the results in Table 3. The interaction terms in Columns (1) and (2) are insignificant, whereas the interaction term is significantly positive in Column (3). This implies that men perform worse than women, and females in only-child families perform worse than females in two-child families.

**Table 3:** Heterogeneity analysis: only children's gender.

	Chinese	Math	English
	(1)	(2)	(3)
Only_child	− 0.621**	− 0.555*	− 0.828***
	(0.280)	(0.290)	(0.255)
Gender (male)	− 5.507***	− 0.632**	− 5.673***
	(0.284)	(0.278)	(0.263)
Only_child $\times$ gender (male)	0.153	− 0.148	0.985***
	(0.351)	(0.350)	(0.332)
Ethnicity (Han)	− 0.491	0.021	− 0.504
	(0.451)	(0.471)	(0.410)
Age	− 2.505	− 4.806***	− 4.464***
	(1.745)	(1.720)	(1.662)
Age_squared	0.066	0.126**	0.118*
	(0.065)	(0.063)	(0.062)
Economics status	Y	Y	Y
Hukou_at_birth	Y	Y	Y
Father education	Y	Y	Y
Mother education	Y	Y	Y
Grade fixed effect	Y	Y	Y
Classroom fixed effect	Y	Y	Y
Constant	83.785***	103.523***	101.568***
	(11.827)	(12.433)	(11.967)
Observations	13,179	13,171	13,170
R^2^	0.182	0.108	0.193

Notes: Standard errors in parentheses, adjusted for within-classroom clustering. *p < 0.10. **p < 0.05. ***p < 0.01.

There are still some confounding factors that should be considered. As^26,27^ explain in substantial detail, this estimation conflates the effects of family size and birth order. Black et al.^26^ find a negative correlation between family size and children's education, but when birth order is included, family size effects become negligible. The birth order effect is strong in long term^27^. They reveal that birth order effects tend to be negative, which would make the estimate of the only-child effect an underestimate. However, this effect is reversed in some contexts. De Haan et al.^28^, using family fixed-effects models, find positive and persistent birth order effects. They suggest that earlier-born children receive less quality time from their mothers and are breastfed for a shorter period. Therefore, we use children from one-child and two-child families as a sample to eliminate the bias caused by birth order effects. We compare the educational outcomes of only children to children from three-child families as well.

Columns (1)–(3) of Table 4 show that children from one-child families perform as well as firstborn children in two-child families in Chinese and English, but children from one-child families perform worse in Mathematics than firstborns in two-child families. This may imply that the significant only-child effect in Table 1 is caused by the outstanding performance of second-born children in two-child families. Columns (4)–(6) of Table 4 show that there is no difference between only children and firstborns in three-child families. The firstborns may be “immune” from the sibship size effect in small families. Our results are consistent with those of Shen et al.^20^.

**Table 4:** The comparison of educational outcomes between only children and firstborns in two-child/three-child families.

	Only children vs. firstborns in two-child families			Only children vs. firstborns in three-child families		
	Chinese	Math	English	Chinese	Math	English
	(1)	(2)	(3)	(4)	(5)	(6)
Only_child	− 0.345	− 0.624**	− 0.212	− 0.667	− 0.527	− 0.929
	(0.261)	(0.261)	(0.247)	(0.500)	(0.606)	(0.570)
Ethnicity (Han)	− 0.649	− 0.286	− 0.598	− 0.503	− 0.700	− 0.579
	(0.478)	(0.492)	(0.436)	(0.523)	(0.579)	(0.518)
Gender (male)	− 5.479***	− 0.759***	− 4.959***	− 5.340***	− 0.723***	− 4.712***
	(0.194)	(0.209)	(0.191)	(0.227)	(0.244)	(0.222)
Age	− 2.785	− 5.084**	− 3.855**	0.951	− 4.178*	− 3.047
	(1.980)	(2.031)	(1.901)	(2.622)	(2.527)	(2.508)
Age_squared	0.076	0.133*	0.096	− 0.066	0.089	0.060
	(0.074)	(0.075)	(0.070)	(0.098)	(0.094)	(0.093)
Economics status	Yes	Yes	Yes	Yes	Yes	Yes
Hukou_at_birth	Yes	Yes	Yes	Yes	Yes	Yes
Father education	Yes	Yes	Yes	Yes	Yes	Yes
Mother education	Yes	Yes	Yes	Yes	Yes	Yes
Grade fixed effect	Yes	Yes	Yes	Yes	Yes	Yes
Classroom fixed effect	Yes	Yes	Yes	Yes	Yes	Yes
Constant	84.344***	108.115***	96.802***	58.775***	101.092***	90.163***
	(13.573)	(13.782)	(13.081)	(17.634)	(17.010)	(16.892)
Observations	10,696	10,689	10,688	8095	8094	8090
R^2^	0.190	0.118	0.197	0.205	0.137	0.210

Notes: Standard errors in parentheses, adjusted for within-classroom clustering. *p < 0.10. **p < 0.05. ***p < 0.01.

Table 5 shows heterogeneous effects by gender. Columns (1)–(3) of Table 5 show that children from female one-child families perform worse than female firstborn children in two-child families in Chinese, Mathematics and English, whereas Columns (4) and (5) show that children from female one-child families perform as well as female firstborn children in three-child families in Chinese and Mathematics. However, children from female one-child families perform worse than female firstborn children in three-child families in English. It is worthy to note that males perform worse than females in the sample of only children and firstborn from two-child and three-child families. We also consider the interaction terms of the results in Table 5. The interaction term in Column (3) is significant, whereas the interaction term is other Columns are insignificant. This analysis shows that females’ performance improves when they have a younger sibling. Their performance changes back when they have a second younger sibling.

**Table 5:** The comparison of educational outcomes between only children and firstborns in two-child/three-child families by genders.

	Only children vs. firstborns in two-child families			Only children vs. firstborns in three-child families		
	Chinese	Math	English	Chinese	Math	English
	(1)	(2)	(3)	(4)	(5)	(6)
Only_child	− 0.561*	− 0.651**	− 0.677**	− 0.483	− 0.448	− 1.234**
	(0.309)	(0.312)	(0.287)	(0.532)	(0.665)	(0.582)
Gender (male)	− 5.824***	− 0.801**	− 5.702***	− 4.559***	− 0.388	− 5.999***
	(0.371)	(0.391)	(0.363)	(1.093)	(1.424)	(1.241)
Only_child $\times$ gender (male)	0.477	0.058	1.027**	− 0.810	− 0.347	1.334
	(0.432)	(0.448)	(0.421)	(1.105)	(1.425)	(1.248)
Ethnicity (Han)	− 0.652	− 0.286	− 0.607	− 0.502	− 0.699	− 0.580
	(0.478)	(0.492)	(0.436)	(0.523)	(0.579)	(0.518)
Age	− 2.791	− 5.085**	− 3.867**	0.957	− 4.176*	− 3.057
	(1.979)	(2.031)	(1.897)	(2.620)	(2.526)	(2.515)
Age_squared	0.076	0.133*	0.097	− 0.066	0.089	0.061
	(0.073)	(0.075)	(0.070)	(0.098)	(0.094)	(0.093)
Economics status	Yes	Yes	Yes	Yes	Yes	Yes
Hukou_at_birth	Yes	Yes	Yes	Yes	Yes	Yes
Father education	Yes	Yes	Yes	Yes	Yes	Yes
Mother education	Yes	Yes	Yes	Yes	Yes	Yes
Grade fixed effect	Yes	Yes	Yes	Yes	Yes	Yes
Classroom fixed effect	Yes	Yes	Yes	Yes	Yes	Yes
Constant	84.514***	108.135***	97.170***	58.605***	101.009***	90.482***
	(13.565)	(13.791)	(13.057)	(17.618)	(17.009)	(16.944)
Observations	10,696	10,689	10,688	8,095	8,094	8,090
R^2^	0.190	0.118	0.197	0.205	0.137	0.210

Notes: Standard errors in parentheses, adjusted for within-classroom clustering. *p < 0.10. **p < 0.05. ***p < 0.01.

In sum, the academic performance of children from one-child families is not better than that of children from two-child families. An only child who is male performs as well as male children from two-child families in English. Our results follow Lao and Dong’s^3^ findings and fill the gap. Lao and Dong^3^ simply compare the average test scores of only children and non-only children, which conflates the effects of family size and birth order. Furthermore, they use the samples whose sibling number is smaller than five, which may enhance the bias from birth order. We compare educational outcomes of only children and children (especially firstborns) in two-child families, which eliminates the bias from birth order.

### Causality

Fertility decisions depend on common factors^29^. Omitted variable bias can make the results of estimation unreliable. To address this problem, we use the gender of the first child to instrument whether a student is in an only child family. Son preference means that parents continue to have children until they have an ideal number of boys. It is a norm deeply rooted in social, cultural, and economic factors in Asian countries such as China, Korea, and India^30^. If parents have son preferences and their first child is a girl, they may have the propensity to try to have another child. Thus, the first child's gender may be a predictor for the probability of being a one-child family.

We use the IV method and estimate a two-stage least squares (2SLS) model. The key is to identify a variable that predicts Only_Child but is uncorrelated with the error term ${\epsilon }_{igc}$ in Eq. (1). We use an indicator for a firstborn girl (first_girl) as an instrument and estimate the 2SLS model:

2 $${Only\_Child}_{igc}={\alpha }_{0}+{\alpha }_{1}\times {first\_girl}_{igc}+X\alpha +Grade+Classroom+{\mu }_{igc}$$

3 $${Score}_{igc}={\pi }_{0}+{\pi }_{1}\times \widehat{{Only\_Child}_{igc}}+X\pi +Grade+Classroom+{v}_{igc}$$

where ${first\_girl}_{igc}$ is a dummy variable that equals 1 if the firstborn is a girl; otherwise, it is 0. This approach is similar in spirit to that of Lee^25^. Lee^25^ first introduced that "first girl" can be a good instrument for family fertility decisions in which strong preferences for sons and small families are social norms. Equations (2) and (3) are the first-stage regression and the second-stage regression, respectively. Standard errors are clustered at the classroom level.

Let us now turn to discussing the validity of the "first girl" as an instrumental variable. If parents controlled births based on sex, the sex of the firstborn cannot be said to be random. Thus, sex-selective abortions may make the instrument invalid. Chinese authorities have been made aware of the misuse of ultrasound machines for prenatal sex determination, which have led to the subsequent abortion of female fetuses. In the Law on Maternal and Infant Health Care, authorities strictly prohibited the use of medical techniques for the identification of fetal sex that may in turn lead to selective abortion for nonmedical reasons. In addition, many studies^31,32^ have found that Chinese parents do not use sex-selective abortions for firstborns, but they may for those of higher birth order. Nie^32^ notes that the policy permits a couple to have a second child if the sex of the firstborn is female. This may serve to dissuade parents from using sex-selective abortion for firstborns. These studies illustrate that the sex of the firstborn is a credible instrumental variable.

Moreover, Kugler and Kumar^33^ point out that the sex of the firstborn is not correlated with educational attainment because it is determined randomly. In other words, the sex of the firstborn will not satisfy the exclusion restriction if it is not random. As discussed in the last paragraph, Chinese parents do not use sex-selective abortion for firstborns. Therefore, Kugler and Kumar^33^ suggest that the sex of the firstborn satisfies the exclusion restriction of the instrument and there is little evidence that households with firstborn girls are different in other ways from those with firstborn boys.

Last, the sex of the firstborn child may be correlated with educational attainment due to the parents’ son preference. Based on the concept of son preference, parents may elect to have one more child if the firstborn is a girl. Parents with a son preference may spend more time on sons, which may lead the only-child effect impact being overestimated when it is positive (and underestimated when it is negative). Thus, we control for students’ gender in the IV estimation.

We also investigate whether the instrument is likely to be exogenous. The results in Table 6 show that the explanatory variables, except for economic status (wealthy) and father's years of schooling, are insignificant. As summarized in Table 1, the proportion of students in wealthy families accounts for 0.2% (26 respondents). Therefore, it has little impact on the only-child effect. This means that the gender of the firstborn is likely exogenous because it is not related to observable family characteristics.

**Table 6:** Regression of firstborn girl on household characteristics.

	(1)	(2)	(3)
	LPM	Probit	Logit
Dep. Var	first_girl	first_girl	first_girl
Ethnicity (Han)	− 0.038	− 0.096	− 0.153
	(0.026)	(0.067)	(0.107)
Economic status (relative poor)	− 0.003	− 0.007	− 0.011
	(0.024)	(0.060)	(0.096)
Economic status (average)	0.032	0.079	0.127
	(0.027)	(0.068)	(0.110)
Economic status (relative rich)	0.045	0.113	0.180
	(0.033)	(0.083)	(0.133)
Economic status (wealthy)	− 0.155*	− 0.400*	− 0.641*
	(0.077)	(0.208)	(0.336)
Hukou_at_birth (non − rural)	− 0.008	− 0.021	− 0.033
	(0.014)	(0.036)	(0.057)
Hukou_at_birth (Resident)	− 0.016	− 0.041	− 0.065
	(0.015)	(0.039)	(0.062)
Hukou_at_birth (other)	− 0.146	− 0.375	− 0.603
	(0.113)	(0.299)	(0.485)
Father's years of schooling	0.003	0.008*	0.012*
	(0.002)	(0.005)	(0.007)
Mother's years of schooling	− 0.003	− 0.007	− 0.010
	(0.002)	(0.005)	(0.008)
Classroom fixed effect	Yes	Yes	Yes
Observations	13,716	13,716	13,716

Notes: Robust standard errors, clustered by classroom, are shown in parentheses. LPM is linear probability model.

Table 7 reports the results of estimating Eqs. (2) and (3) for the standardized scores of Chinese, mathematics, and English. The IVs in the first-stage regression have significant explanatory power. The first girl significantly decreases the students' probability of being only children. All the F-statistic values for the instrument are much larger than the rule-of-thumb threshold of 10^34^. The results, except English, are significantly negative. The IV estimates show that the OLS estimates are underestimated, possibly due to omitted factors.

**Table 7:** The IV estimation of the effect of only-child status on standardized test scores.

	Chinese	Math	English
	(1)	(2)	(3)
**Second stage: dependent variable is student's test score**			
Only_child	− 2.435*	− 2.924**	− 0.085
	(1.376)	(1.376)	(1.440)
Ethnicity (Han)	− 0.313	0.470	− 0.526
	(0.540)	(0.597)	(0.518)
Gender (male)	− 5.343***	− 0.527*	− 5.397***
	(0.294)	(0.282)	(0.262)
Age	− 5.679***	− 8.282***	− 7.346***
	(1.921)	(1.912)	(1.843)
Age_squared	0.178**	0.246***	0.220***
	(0.071)	(0.069)	(0.067)
**First stage: dependent variable is whether a student is an only-child**			
First_girl	− 0.213***	− 0.213***	− 0.215***
	(0.018)	(0.018)	(0.018)
Economics status	Yes	Yes	Yes
Hukou_at_birth	Yes	Yes	Yes
Father education	Yes	Yes	Yes
Mother education	Yes	Yes	Yes
Grade fixed effect	Yes	Yes	Yes
Classroom fixed effect	Yes	Yes	Yes
f-stat of excl. instrument	142.41	141.93	139.92
Observations	13,179	13,171	13,170

Notes: Standard errors in parentheses, adjusted for within-classroom clustering. *p < 0.10. **p < 0.05. ***p < 0.01.

The IV estimation is called the Local Average Treatment Effect (LATE). The change in the instrumental variable causes the endogenous variable to have different probabilities in different values in the first stage, and this difference is then transferred to the dependent variable in the second stage^35^ (Abadie 2003^36^). Imbens^35^, and Abadie (2003)^36^ divide the population into four categories: always-takers, defiers, compliers, and never-takers. In other words, LATE can only be identified by estimating compliers. Thus, we estimate the only-child effect using compliers as a sample. In our case, the gender of the firstborn is an instrumental variable. When the first child of a family is a girl, parents have a higher likelihood of having one more child. Thus, we define families with an only girl as defiers, whereas two-child families with a male firstborn as always-takers and never-takers.In our case, it is difficult to identify always-takers and never-takers. We cannot know the exact fertility motivation of two-child families with male firstborns. We remove defiers, always-takers, and never-takers and define the rest of the sample as compliers.

Table 8 shows an estimation of the only-child effect using compliers as a sample. The results of Table 8 are consistent with the results of Table 2. The only-child effects are significant in the subjects of Chinese and mathematics, but they are insignificant in English. Specifically, children in one-child families on average have lower (0.96 and 1.06) standard scores in Chinese and mathematics, respectively, than children in two-child families.

**Table 8:** The analysis using compliers as a sample.

	Chinese	Math	English
	(1)	(2)	(3)
only_child	− 0.967**	− 1.066***	− 0.255
	(0.378)	(0.377)	(0.382)
Ethnicity (Han)	− 0.219	0.148	− 0.533
	(0.631)	(0.693)	(0.625)
Gender (male)	− 5.150***	− 0.412	− 5.430***
	(0.386)	(0.371)	(0.368)
Age	− 0.556	− 5.346**	− 5.297**
	(2.659)	(2.529)	(2.379)
Age_squared	− 0.012	0.144	0.140
	(0.099)	(0.093)	(0.088)
Economics Status	Yes	Yes	Yes
Hukou_at_birth	Yes	Yes	Yes
Father Education	Yes	Yes	Yes
Mother Education	Yes	Yes	Yes
Grade Fixed Effect	Yes	Yes	Yes
Classroom Fixed Effect	Yes	Yes	Yes
Observations	7559	7553	7555
R^2^	0.190	0.133	0.195

Notes: Standard errors in parentheses, adjusted for within-classroom clustering. *p < 0.10. **p < 0.05. ***p < 0.01.

Table 9 shows the estimation of the effect of only-child status on standardized test scores by gender. We use the male and female subsamples with IV estimation. The IVs in the first-stage regression have significant explanatory power. The first girl significantly decreases the students' probability of being only children. All the F-statistic values for the instrument are much larger than the rule-of-thumb threshold of 10^34^. The results of the male subsample are consistent with Table 6. Only-child effects are significant in the subjects of Chinese and mathematics, while these effects do not work in female subsamples.

**Table 9:** The IV estimation of the effect of only-child status on standardized test scores by genders.

	Female	Male	Female	Male	Female	Male
	Chinese	Chinese	Math	Math	English	English
	(1)	(2)	(3)	(4)	(5)	(6)
**Second stage: dependent variable is student's test score**						
Only_child	− 0.430	− 1.420**	0.232	− 1.319**	− 0.979	− 0.555
	(0.981)	(0.612)	(1.147)	(0.600)	(1.045)	(0.642)
Ethnicity (Han)	0.168	− 0.976	1.424**	− 1.234**	− 0.197	− 0.699
	(0.548)	(0.663)	(0.700)	(0.599)	(0.529)	(0.635)
Age	− 0.643	− 4.114	− 1.185	− 7.407***	− 1.028	− 7.323***
	(2.080)	(2.577)	(2.382)	(2.245)	(2.062)	(2.457)
Age_squared	− 0.000	0.121	− 0.008	0.220***	− 0.000	0.215**
	(0.076)	(0.095)	(0.088)	(0.082)	(0.076)	(0.090)
**First stage: dependent variable is whether a student is an only-child**						
First_girl	− 0.381***	− 0.562***	− 0.382***	− 0.562***	− 0.381***	− 0.563***
	(0.019)	(0.018)	(0.014)	(0.018)	(0.020)	(0.018)
Economics status	Yes	Yes	Yes	Yes	Yes	Yes
Hukou_at_birth	Yes	Yes	Yes	Yes	Yes	Yes
Father education	Yes	Yes	Yes	Yes	Yes	Yes
Mother education	Yes	Yes	Yes	Yes	Yes	Yes
Grade Fixed effect	Yes	Yes	Yes	Yes	Yes	Yes
Classroom fixed effect	Yes	Yes	Yes	Yes	Yes	Yes
f-stat of excl. instrument	364.40	952.81	363.91	950.25	362.41	954.94
Observations	6241	6938	6237	6934	6235	6935

Notes: Standard errors in parentheses, adjusted for within-classroom clustering. *p < 0.10. **p < 0.05. ***p < 0.01.

In summary, only-child status has causal effect on scores in Chinese and mathematics. Male children in one-child families perform worse in Chinese and mathematics than males in two-child families. Finally, only-child effects do not work in female subsamples.

### Mechanism check and mediation analysis

We find that an only-child status has significantly negative effects on students’ Chinese and mathematics performance. Shen et al.^20^ mentioned that the sibship size effect depends on the relative magnitude of two competing effects: scale economies and resource dilution. In this section, we explore potential mechanisms and, in particular, focus on economies of scale (including willpower and extraversion) and resource dilution effects (including parental control and parental support), which may change when an additional child is added to a one-child family. Accordingly, we conduct a decomposition analysis, which shows that these channels can explain the only-child effect.

### Economies of scale

Here, we examine how economies of scale vary by the only-child status of a child.

The increase in sibship size may benefit children in the family by reducing the average cost of each child, which is referred to as economies of scale. The economy of scale affects children’s quality in two ways. First, parents can practice and improve their skills when the family size increases. They know how the shape their children's personalities efficiently. Second, children can play with their siblings, which reduces the cost to search for peers. Children are forced to develop interpersonal skills in the presence of siblings^37,38^, and they learn self-control as well^39^. In fact, Cameron et al.^40^ claim that “sibling deprivation” results in only children in fewer skills for peer-based interactions, which increase the cost of acquiring social interaction. It seems that the increase in sibship size reduces the costs of children’s development.

However, it is difficult to measure all costs of raising children. Some costs are unobservable. For example, we cannot measure the opportunity cost of interacting with siblings. Therefore, we assume that children with low costs are easy to acquire a better quality of personality. We use willpower and extraversion as proxies for economies of scale.

First, it is possible that children from one-child families may have an average lower level of willpower, which, in turn, affects student outcomes. To assess the relevance of this mechanism, we construct an index using principal component analysis (PCA) and three items from the student survey: "I still go to school even if I feel a little uncomfortable or have any absent excuses", "I will try my best to finish my homework even if I do not like it", and "I will try my best to finish my homework even if I have to spend a great deal of time on it". Students are asked to rate to what extent they agree with the statement on a scale from 1 (strongly disagree) to 4 (strongly agree). The Cronbach’s alpha of the index is 0.728.

Table 10 reports the estimation results, showing that the willpower of children in one-child families is on average lower than that of children in two-child families. Zhang and Qian^41^ and Yang et al.^42^ found that children from one-child families have less self-control. The results of this paper are consistent with their findings. Furthermore, Tangney, Baumeister, and Boone^43^ suggest that a higher self-control level was associated with higher academic performance. They use college students as a sample and found that students with higher scores on self-control had better grade point averages. This paper supports their opinion. We find that junior high school students' willpower is correlated with their educational attainment. Table 9 shows that the coefficients of willpower to Chinese and mathematics are 0.95 and 1.11, respectively. This means that students with higher level of willpower have better academic performance. The only-child status can indirectly affect educational attainment through a mediating mechanism—significantly lowering willpower levels. In particular, 8.18% of the total effect on success in Chinese is mediated by willpower, whereas 8.22% of the total effect on mathematics is mediated by willpower.

**Table 10:** Mechanism: Willpower.

	Chinese	Willpower	Chinese	Math	Willpower	Math
	(1)	(2)	(3)	(4)	(5)	(6)
Only_child	− 0.056***	− 0.048**	− 0.514***	− 0.648***	− 0.048**	− 0.595***
	(0.198)	(0.021)	(0.197)	(0.207)	(0.021)	(0.206)
Willpower			0.950***			1.112***
			(0.082)			(0.086)
Controls	Yes	Yes	Yes	Yes	Yes	Yes
Economics status	Yes	Yes	Yes	Yes	Yes	Yes
Hukou_at_birth	Yes	Yes	Yes	Yes	Yes	Yes
Father education	Yes	Yes	Yes	Yes	Yes	Yes
Mother education	Yes	Yes	Yes	Yes	Yes	Yes
Grade fixed effect	Yes	Yes	Yes	Yes	Yes	Yes
Classroom fixed effect	Yes	Yes	Yes	Yes	Yes	Yes
Indirect effect			− 0.045**			− 0.050**
p-value			0.027			0.026
% of mediated			8.18%			8.22%
Observations	12,726	12,726	12,726	12,719	12,719	12,719

Notes: Standard errors in parentheses, adjusted for within-classroom clustering. *p < 0.10. **p < 0.05. ***p < 0.01.

Some explanations of the Chinese case are as follows. Children in one-child families are more likely to be spoiled. Furnham and Wu^44^ explain that parents may invest more financial and nonfinancial resources in their only-child, particularly boys. Parents may excessively dote on their only child. They may protect their only child from "normal" challenges, weakening the willpower of the child. Moreover, self-control predicts academic performance at all levels of schooling^45^, including middle school^46,47^. It improves homework completion and classroom conduct^48^. This may be the explanation of willpower as a mediator of the only-child effect on educational attainment.

Second, it is possible that extraversion level of children in one-child families may be lower on average, which affects student outcomes. To assess the relevance of this mechanism, we construct an index using PCA and three items from the student survey: "I consider myself easy to get along with", "I feel close to people in this school", and "I often take part in school/class activities". Students are asked to rate to what extent they agree with the statement on a scale from 1 (strongly disagree) to 4 (strongly agree). The Cronbach’s alpha of the index is 0.711.

Table 11 reports the estimation results, which show that the extraversion of children in one-child families is on average lower than that of children in two-child families. Children in two-child families have a higher rate of interaction with others (such as a sibling), which may lead to the children in two-child families developing a higher level of extraversion. Extraversion has been linked with academic success in school. For example, students with high extraversion are likely to invest their efforts in intellectual activities^49^,therefore, there is a positive relationship between cognitive ability and extraversion^50^. Moreover, Steel et al.^51^ suggest that the sociability component of extraversion help students learn due to frequent interactions with teachers. Teachers have a tendency to perceive talkative children as more intelligent and more academically gifted than shy students^52^. Our results are consistent with their opinion. Table 10 shows that the coefficients of extraversion to Chinese and mathematics are 1.07 and 1.05, respectively. This means that students with higher-level willpower have better academic performance. A total of 12.48% of the total effect on Chinese is mediated by extraversion, whereas 10.44% of the total effect on mathematics is mediated by extraversion.

**Table 11:** Mechanism: Extraversion.

	Chinese	Extraversion	Chinese	Math	Extraversion	Math
	(1)	(2)	(3)	(4)	(5)	(6)
only_child	− 0.546***	− 0.063***	− 0.478***	− 0.642***	− 0.063***	− 0.575***
	(0.197)	(0.020)	(0.196)	(0.205)	(0.020)	(0.204)
extraversion			1.072***			1.059***
			(0.085)			(0.089)
Controls	Yes	Yes	Yes	Yes	Yes	Yes
Economics status	Yes	Yes	Yes	Yes	Yes	Yes
Hukou_at_birth	Yes	Yes	Yes	Yes	Yes	Yes
Father education	Yes	Yes	Yes	Yes	Yes	Yes
Mother education	Yes	Yes	Yes	Yes	Yes	Yes
Grade fixed effect	Yes	Yes	Yes	Yes	Yes	Yes
Classroom fixed effect	Yes	Yes	Yes	Yes	Yes	Yes
Indirect effect			− 0.068***			− 0.067***
p-value			0.002			0.002
% of mediated			12.48%			10.44%
Observations	12,921	12,921	12,921	12,913	12,913	12,913

Notes: Standard errors in parentheses, adjusted for within-classroom clustering. * *p* < 0.10. **p < 0.05. ***p < 0.01.

In our case, the expansion of family size has a positive effect on children’s development. An additional sibling may help them improve their personalities (in willpower and extraversion). We suggest that is the above improvement may be attributed to scale economy effect.

### Resource dilution

A second possible mechanism is the resource dilution effect. Parents may be strict with their only children and likely to increase parental control and parental support because they are investing more in their only child. Parental control is defined as parental intrusiveness, pressure, or domination intended to control children's behavior^53^, whereas parental support can be defined as the value of parental assistance. Pomerantz et al.^54^ found that parental control decreased children's efforts in challenging learning situations and lowered their sense of competence and autonomy.

We explored three items about strictness of parents to measure parental control. These included "how strict are your parents concerning your homework and exams", "how strict your parents are with your school performance", "how strict are your parents concerning your school activities", "how strict are your parents regarding curfew", "how strict are your parents regarding your time using the Internet", and "how strict are your parents regarding your time spent watching television". The numerical value 1–3 represents the parents' strictness from low to high. The Cronbach’s alpha of the index is 0.731. Table 12 reports the estimation results. The results show that parental control of children in one-child families is on average lower than that of children in two-child families and is not correlated to children’s outcomes. It is interesting that the average parental control level of only children is lower than that of children in two-child families. This seems to be the net effect of the little emperor. Families do what the child wants, not the other way around. Only children are more likely to be spoiled.

**Table 12:** Mechanism: Parental control.

	Chinese	Parental control	Chinese	Math	Parental control	Math
	(1)	(2)	(3)	(4)	(5)	(6)
Only_child	− 0.521***	− 0.075***	− 0.514***	− 0.562***	− 0.076***	− 0.556***
	(0.197)	(0.021)	(0.197)	(0.205)	(0.021)	(0.205)
Parental control			0.091			0.088
			(0.082)			(0.086)
Controls	Yes	Yes	Yes	Yes	Yes	Yes
Economics status	Yes	Yes	Yes	Yes	Yes	Yes
Hukou_at_birth	Yes	Yes	Yes	Yes	Yes	Yes
Father education	Yes	Yes	Yes	Yes	Yes	Yes
Mother education	Yes	Yes	Yes	Yes	Yes	Yes
Grade fixed effect	Yes	Yes	Yes	Yes	Yes	Yes
Classroom fixed effect	Yes	Yes	Yes	Yes	Yes	Yes
Indirect effect			− 0.006			− 0.006
*p*-value			0.270			0.308
% of mediated						
Observations	12,976	12,976	12,976	12,968	12,968	12,968

Notes: Standard errors in parentheses, adjusted for within-classroom clustering. * *p* < 0.10. **p < 0.05. ***p < 0.01.

Finally, we explored two items about parental support. They are "how frequently do your parents check your homework and assignment" (check) and "how frequently do your parents tutor you while working on homework and assignments" (tutor). The numerical value 1–4 represents "never", "1–2 days per week", "3–4 days per week ", and "everyday". The Cronbach’s alpha of the index is 0.761. Table 13 reports the estimation results, which show that the parental support of children in one-child families di not differ from that of children in two-child families.

**Table 13:** Mechanism: Parental support.

	Chinese	Parental support	Chinese	Math	Parental support	Math
	(1)	(2)	(3)	(4)	(5)	(6)
Only_child	− 0.514***	0.028	− 0.492**	− 0.581***	0.028	− 0.553***
	(0.197)	(0.020)	(0.197)	(0.206)	(0.020)	(0.205)
Parental support			− 0.787			− 0.996***
			(0.086)			(0.090)
Controls	Yes	Yes	Yes	Yes	Yes	Yes
Economics status	Yes	Yes	Yes	Yes	Yes	Yes
Hukou_at_birth	Yes	Yes	Yes	Yes	Yes	Yes
Father education	Yes	Yes	Yes	Yes	Yes	Yes
Mother education	Yes	Yes	Yes	Yes	Yes	Yes
Grade fixed effect	Yes	Yes	Yes	Yes	Yes	Yes
Classroom fixed effect	Yes	Yes	Yes	Yes	Yes	Yes
Indirect effect			− 0.022			− 0.028
p-value			0.161			0.167
% of mediated						
Observations	12,959	12,959	12,959	12,951	12,951	12,951

Notes: Standard errors in parentheses, adjusted for within-classroom clustering. * *p* < 0.10. **p < 0.05. ***p < 0.01.

Tables 12 and 13 show that parental control and parental support are not mediators in the only-child effect. Thus, the resource dilution effect does not work in our study. Shen et al.^20^ suggested that the scale economies effect was strong, and the resource dilution effect was weak with a low number of siblings. Our results support their opinions.

Finally, we discuss the overlap between economies of scale and resource dilution. Table 11 shows that there are differences in parental control between only children and children in two-child families. Less controlling parents can have more extroverted children. Children may develop their conscientiousness when parental control is absent. Table 14 shows that parental control is correlated positively to willpower and extraversion. It may imply that parental control helps the personality development of children. Since children in two-child families have a higher level of control from parents, they have a high level of willpower and extraversion, which benefit educational performance. In this opinion, willpower and extraversion are not only proxies of economy scale but also proxies of resource dilution.

**Table 14:** The association between parental control and children’s personalities.

	Willpower	Extraversion
	(1)	(2)
Parental control	0.147***	0.166***
	(0.010)	(0.010)
Controls	Yes	Yes
Economics status	Yes	Yes
Hukou_at_birth	Yes	Yes
Father education	Yes	Yes
Mother education	Yes	Yes
Grade fixed effect	Yes	Yes
Classroom fixed effect	Yes	Yes
Observations	12,801	12,999
R^2^	0.135	0.175

However, the evidence of this paper is insufficient to support the above argument. First, the correlation does not eliminate omitted factors. There are some factors affecting both parental control and children’s personalities. For instance, the community and neighborhood may strengthen both parental control and children’s personalities. Second, there is simultaneity in the correlation between parental control and children’s personalities. Parents change their control over children when children’s personalities change. Thus, we suggest that there may be an overlap between economies of scale and resource dilution but we cannot claim that the overlap does exist. It can be explored in the future studies.

## Conclusion and discussion

The Chinese government abolished the only-child policy in 2015, and it has encouraged increased fertility since 2015. The impact on existing children's educational attainment may be one of the most important factors in parents' fertility decisions. This paper provides evidence that the average test scores of children in one-child families are lower than those of children in two-child families. This implies that the marginal effect o sibship size on the educational performance of children in one-child families is nonnegative.

The study of quantity-quality trade-off is well established (e.g.,^5^. Lao and Dong^3^ found that only children's educational outcomes are significantly higher than the educational outcomes of children having siblings. Using the same dataset of this paper, they compared children in one-child families to children having more than one sibling but fewer than five, and found that the sibship effect on educational attainment may be overestimated. They neglect the scale economies effect, which is one of the contributions of this paper, as we found that the scale economies effect is dominant to the sibship size effect when the family size is small.

This article uses a sample of students from one-child families and two-child families. It provides evidence that an additional sibling is not harmful to the educational attainment of the original only child. This result is similar to Shen et al.^20^, Diaz and Fiel's study^17^ and has practical use for China's policymaking. Although Lao and Dong^3^ introduce personalities to analyze the only-child effect, the large family size causes some bias. For example, they suggest that the extraversion of children in one-child families is higher than that of children in large families,interestingly, the "middle child syndrome" may lead to a positive effect. Since children in large families have more opportunities to play with others (their sibling), their level of extraversion should be greater than that of an only child. This paper uses a sample of students from one-child families and two-child families to eliminate the "middle child syndrome" bias. Furthermore, Lao and Dong^3^ neglect the role of willpower in the only-child effect. We fill this gap and find that willpower is an important influencing factor to the negative only-child effect.

Scholars illustrate the quantity-quality tradeoff by the resource dilution hypothesis (e.g.,^55^. They suggest that the arrival of newborns dilute a family's financial resources. However, Lao and Dong^3^ pointed out that some resources can be shared (such as books). Although Lao and Dong^3^ admited that the resource dilution effect was an important channel of the sibship size effect, our results find that parental concerns are the same in both one-child families and two-child families. Therefore, there may be little or no dilution effect of resources for small families. Furthermore, economies of scale regarding raising more children may offset parts of the dilution effect. Both parents' experiences of raising older children and effective sibling interactions may also weaken the dilution effect^15,21^. Thus, the effect of sibship size on educational performance may be an inverted U shape. Since the marginal sibship size effect for an only child is nonnegative, a "two-child policy" may not be harmful to the educational attainment of children.

There are some limitations of these data. First, this data cannot identify the firstborn's gender in three-child families. Second, the sample size of three-child families is too small (approximately 1531 observations) compared to the sample size of one-child and two-child families. Both limitations above make further studies on three-child families difficult. Finally, the dataset is based on schools and classrooms and not just family. Thus, we cannot control for family fixed effects using this dataset. Although the "three-child policy" has been implemented since May 31st, 2021, it is still worth further studying the sibship effect on educational attainment for one-child families due to the current low fertility desire of China.

### Ethical approval

All methods were performed in accordance with relevant guidelines and regulations.

## Supplementary Information

Supplementary Information.

## Data Availability

The data that support the findings of this study are available from Renmin University of China. Restrictions apply to the availability of these data, which were used under license for this study. Data are available from Yehui Lao with the permission of Renmin University of China. The data that support the findings of this study are available from Renmin University of China but restrictions apply to the availability of these data, which were used under license for the current study, and so are not publicly available. Data are however available from the authors upon reasonable request and with permission of Renmin University of China. Readers are able to download freely from http://www.cnsda.org/index.php?r=projects/view&id=72810330
