# Supplementary material for: Comparison of educational performance between the only children and children in two-child families
Source: Sci Rep. 2022 Sep 12;12:15355. doi: 10.1038/s41598-022-19730-3 (PMC9468154; doi:10.1038/s41598-022-19730-3)
Supplement: Supplementary file 1 — Supplementary Information. [file 41598_2022_19730_MOESM1_ESM.docx]

## Appendix

Table A1

|  | Chinese | only_childd | grade9 | ethnicity (Han) | gender(male) | age | age_2 | econ | fa_eduy | mo_eduy |
| --- | --- | --- | --- | --- | --- | --- | --- | --- | --- | --- |
| Chinese | 1 |  |  |  |  |  |  |  |  |  |
| only_child | -0.0204 | 1 |  |  |  |  |  |  |  |  |
| grade9 | -0.01 | 0.0413 | 1 |  |  |  |  |  |  |  |
| ethnicity (Han) | -0.0193 | 0.0438 | 0.0084 | 1 |  |  |  |  |  |  |
| gender(male) | -0.2947 | 0.0455 | -0.0149 | 0.0136 | 1 |  |  |  |  |  |
| age | -0.0579 | -0.0479 | 0.8293 | -0.0565 | 0.049 | 1 |  |  |  |  |
| age_2 | -0.0573 | -0.0505 | 0.825 | -0.0586 | 0.0486 | 0.9989 | 1 |  |  |  |
| econ | 0.0329 | 0.1823 | -0.0211 | 0.0587 | -0.0452 | -0.0902 | -0.0926 | 1 |  |  |
| fa_eduy | 0.1026 | 0.3395 | -0.0224 | 0.0237 | -0.0428 | -0.13 | -0.1323 | 0.2536 | 1 |  |
| mo_eduy | 0.077 | 0.3715 | -0.0298 | 0.0714 | -0.0389 | -0.1532 | -0.1569 | 0.278 | 0.6755 | 1 |

Table A2

|  | Math | only_childd | grade9 | ethnicity (Han) | gender(male) | age | age_2 | econ | fa_eduy | mo_eduy |
| --- | --- | --- | --- | --- | --- | --- | --- | --- | --- | --- |
| Math | 1 |  |  |  |  |  |  |  |  |  |
| only_child | -0.0066 | 1 |  |  |  |  |  |  |  |  |
| grade9 | 0 | 0.0413 | 1 |  |  |  |  |  |  |  |
| ethnicity (Han) | -0.0059 | 0.0436 | 0.0092 | 1 |  |  |  |  |  |  |
| gender(male) | -0.0609 | 0.0455 | -0.0148 | 0.0139 | 1 |  |  |  |  |  |
| age | -0.0578 | -0.0476 | 0.8294 | -0.0553 | 0.0488 | 1 |  |  |  |  |
| age_2 | -0.0571 | -0.0502 | 0.8251 | -0.0574 | 0.0485 | 0.9989 | 1 |  |  |  |
| econ | 0.0115 | 0.182 | -0.0214 | 0.0576 | -0.0448 | -0.0901 | -0.0926 | 1 |  |  |
| fa_eduy | 0.0974 | 0.3393 | -0.0226 | 0.0225 | -0.0427 | -0.1297 | -0.132 | 0.2535 | 1 |  |
| mo_eduy | 0.0756 | 0.3712 | -0.0306 | 0.0708 | -0.039 | -0.1537 | -0.1574 | 0.2779 | 0.6758 | 1 |

Table A3

|  | English | only_childd | grade9 | ethnicity (Han) | gender(male) | age | age_2 | econ | fa_eduy | mo_eduy |
| --- | --- | --- | --- | --- | --- | --- | --- | --- | --- | --- |
| English | 1 |  |  |  |  |  |  |  |  |  |
| only_child | -0.0002 | 1 |  |  |  |  |  |  |  |  |
| grade9 | -0.0117 | 0.0413 | 1 |  |  |  |  |  |  |  |
| ethnicity (Han) | -0.0131 | 0.0435 | 0.0084 | 1 |  |  |  |  |  |  |
| gender(male) | -0.2847 | 0.0452 | -0.0152 | 0.0133 | 1 |  |  |  |  |  |
| age | -0.0768 | -0.0482 | 0.8295 | -0.0567 | 0.0484 | 1 |  |  |  |  |
| age_2 | -0.0761 | -0.0508 | 0.8253 | -0.0588 | 0.0481 | 0.9989 | 1 |  |  |  |
| econ | 0.0286 | 0.1817 | -0.0213 | 0.059 | -0.045 | -0.0908 | -0.0933 | 1 |  |  |
| fa_eduy | 0.1164 | 0.3394 | -0.0229 | 0.0235 | -0.0427 | -0.1303 | -0.1326 | 0.2539 | 1 |  |
| mo_eduy | 0.0989 | 0.3715 | -0.0303 | 0.0716 | -0.0388 | -0.1536 | -0.1573 | 0.2777 | 0.676 | 1 |
